# Supplementary material for: The Molecular Basis for Selectivity of the Cytotoxic Response of Lung Adenocarcinoma Cells to Cold Atmospheric Plasma
Source: Biomolecules. 2023 Nov 20;13(11):1672. doi: 10.3390/biom13111672 (PMC10669024; doi:10.3390/biom13111672)

Table S1 – Characteristics of the sequenced samples

Cell Line	Time after CAP Treatment, h	Sample Name	Number of Replicates	Number of Sequenced Reads, millions
A549	0 (control)	A1	2	21.13
		A2		
	3	A3	2	22.62
		A4		
	24	A7	2	21.48
		A8		
Wi-38	0 (control)	W1	2	23.57
		W2		
	3	W3	2	24.53
		W4		
	24	W7	2	24.91
		W8		

Table S2 – Common terms enriched by the genes which are differentially regulated in the response of A549 cells to CAP treatment. The table includes selected results of the Enrichr analysis of the top 250 transcripts that are upregulated (Up) or downregulated (Down) 3 h and 24 h after CAP treatment (Enrichr terms of "MSigDB Hallmark 2020"; "GO Biologic Process 2021"; "Panther 2016"; and "KEGG 2021 Human" libraries).

Type of Regulation	Selected Terms	Representative Genes	Number of Genes
Up	Apoptosis	*JUN;CDKN1A;BTG2;GADD45B;GADD45A;EMP1;BCL10;ISG20;BCL2L11;DDIT3*	48
	Endoplasmic reticulum lumen (GO:0005788)	*COLGALT2;ADAMTSL4;COL6A3;PTGS2;COL1A1;COL1A2;COL16A1;SDC2;COL7A1;FAM20C*	14
	Hypoxia	*PPP1R15A;ERRFI1;CDKN1A;BTG1;SDC4;CITED2;STC1;TNFAIP3;ADM;IRS2*	49
	KRAS Signaling Up	*PPP1R15A;EMP1;TNFAIP3;PRDM1;INHBA;PTGS2;KLF4;DUSP6;RELN;MAFB*	25
	MAPK signaling pathway	*DUSP5;JUN;GADD45B;GADD45A;RASGRF1;ARRB1;FOS;DUSP8;DUSP6;MAPK13*	28
	Negative regulation of cellular macromolecule biosynthetic process (GO:2000113)	*HDAC5;CDKN1A;BTG2;CITED2;NPAS1;TOB1;HHEX;LBH;NFIL3;IFI16*	44

	p53 Pathway	*PPP1R15A;CDKN1A;BTG2;BTG1;RNF19B;PPM1D;TOB1;FDXR;HMOX1;IER5*	51
	Regulation of apoptotic process (GO:0042981)	*CITED2;BCL10;BCL2L11;ADAMTSL4;TP53INP1;PMAIP1;SLIT2;ZNF420;PLK3;JUN*	52
	Regulation of cell population proliferation (GO:0042127)	*BEX4;PTPRU;CDKN1A;BTG2;BTG1;CXCL8;IRS2;PPM1D;TOB1;EFNB2*	56
	Response to endoplasmic reticulum stress (GO:0034976)	*PPP1R15A;JUN;BCL2L11;CXCL8;DDIT3;SELENOS;CHAC1;ATF3;BBC3;ATF4*	18
	TNF-alpha Signaling via NF-kB	*PPP1R15A;BTG2;CDKN1A;BTG1;SDC4;RNF19B;TNFAIP3;IRS2;PTGS2;SOCS3*	80
	UV Response Up	*BTG2;BTG1;FOS;AQP3;NR4A1;NXF1;BCL2L11;CA2;HMOX1;FOSB*	27
Down	E2F Targets	*TOP2A;CDCA3;PRKDC;BUB1B;HMMR;MKI67;SMC4;LMNB1;AURKA;CDC20*	29
	G2-M Checkpoint	*TOP2A;CCNF;TTK;KIF11;HMMR;MKI67;SMC4;CENPA;KIF15;LMNB1*	34
	Microtubule cytoskeleton organization involved in mitosis (GO:1902850)	*STIL;PLK1;BUB1B;TTK;KNL1;KIF11;CENPA;NDC80;AURKA;CDC20*	25
	Mitotic sister chromatid segregation (GO:0000070)	*PLK1;KIF14;NCAPG;SMC4;KNSTRN;NDC80;SGO2;CENPE;CCNB1;KIF18A*	20
	Mitotic Spindle	*TOP2A;PIF1;TTK;KIF11;SMC4;KIF15;LMNB1;AURKA;EPB41L2;KNTC1*	32
	Mitotic spindle organization (GO:0007052)	*STIL;PLK1;BUB1B;TTK;KNL1;KIF11;CENPA;NDC80;AURKA;CDC20*	28
	mTORC1 Signaling	*DHFR;SCD;CCNF;PLK1;ELOVL6;DHCR24;BUB1;ACACA;AURKA;DDIT4*	12
	Regulation of cell cycle process (GO:0010564)	*BORA;PRC1;PLK1;KIF20A;KIF11;MKI67;KIF20B;KNSTRN;BUB1;AURKA*	13

Table S3 – Common terms enriched by the genes which are differentially regulated in the response of Wi-38 cells to CAP treatment. The table includes selected results of the Enrichr analysis of the top 250 transcripts that are upregulated (Up) or downregulated (Down) 3 h and 24 h after CAP treatment (Enrichr terms of "MSigDB Hallmark 2020"; "GO Biologic Process 2021"; "Panther 2016"; and "KEGG 2021 Human" libraries).

Type of Regulation	Selected Terms	Representative Genes	Number of Genes
Up	Apoptosis	*CDKN1A;BTG2;RETSAT;GADD45A;DDIT3;FDXR;TAP1;HMOX1;FAS;SAT1*	45
	DNA damage response, signal transduction by p53 class mediator (GO:0030330)	*ACER2;PIDD1;PLK3;BTG2;CDKN1A;GADD45A;SESN2;MDM2;SFN;ZNF385A*	14
	Hypoxia	*CDKN1A;DUSP1;DDIT3;BHLHE40;HMOX1;TNFAIP3;S100A4;ANGPTL4;PPP1R15A;ERRFI1*	29
	Intrinsic apoptotic signaling pathway (GO:0097193)	*ERN1;CDKN1A;DDIT3;DAB2IP;TNFRSF10B;SFN;HIC1;EPHA2;BBC3;PPP1R15A*	15
	KRAS Signaling Up	*RELN;FUCA1;ITGA2;SEMA3B;TNFAIP3;ANGPTL4;EREG;PPP1R15A;LIF;PLAUR*	23
	Mitotic G1 DNA damage checkpoint signaling (GO:0031571)	*PIDD1;PLK3;BTG2;CDKN1A;GADD45A;MDM2;SFN;ZNF385A;E2F7;GADD45A*	12
	Myc Targets V2	*RCL1;NOP16;PUS1;GRWD1;MRTO4;RRP12;PPRC1;RRP9;SRM*	9
	p53 Pathway	*CYFIP2;CDKN1A;BTG2;RETSAT;CD82;ABHD4;PVT1;DGKA;RNF19B;SLC3A2*	58
	Regulation of apoptotic process (GO:0042981)	*PLK3;GADD45A;SPHK1;OSGIN1;DAB2IP;FHL2;TNFRSF10B;ACER2;PIDD1;NUAK2*	54
	Regulation of cell population proliferation (GO:0042127)	*PTPRU;BTG2;CDKN1A;JAG1;CSF1;KSR1;INSR;OSGIN1;DAB2IP;PPM1D*	55

	TGF-beta Signaling	*PMEPA1;LTBP2;SMAD7;CDKN1A;SMAD7;BCAR3;KLF10;PPP1R15A;SERPINE1;PMEPA1*	20
	TNF-alpha Signaling via NF-kB	*BTG2;CDKN1A;JAG1;CSF1;GADD45A;DUSP1;SPHK1;RNF19B;TAP1;TNFAIP3*	65
	UV Response Dn	*DUSP1;BHLHE40;FHL2;SMAD7;DUSP1;INSIG1;DLC1;SERPINE1;F3;IGF1R*	13
	Xenobiotic Metabolism	*CYFIP2;HES6;RETSAT;SMOX;CYP4F2;HMOX1;FAS;XDH;EPHA2;MT2A*	13
Down	Cell cycle	*CCNB1;DBF4;CDKN2C;CCNE2;MCM6;SKP2;BUB1;CDKN1B;PRKDC;ATM*	11
	Cyclin-dependent protein kinase holoenzyme complex (GO:0000307)	*CCNB1;CCNE2;CCNG2*	3
	Cytoskeleton (GO:0005856)	*ANLN;DENND2A;MTUS1;RHOBTB3;ALDOC;KLHL4;HMMR;ADD3;RHOBTB1;AURKA*	28
	E2F Targets	*TOP2A;CDKN2C;ANP32E;DEPDC1;HMMR;MCM6;LBR;DCK;DLGAP5;LMNB1*	15
	Endoplasmic reticulum lumen (GO:0005788)	*ERO1A;COL3A1;P4HA1;COL5A1;PDGFD;COL11A1;COL8A1;UGGT2;CHRDL1;FSTL1*	23
	Endoplasmic reticulum membrane (GO:0005789)	*ERO1A;MOXD1;ARL6IP1;PIGN;INSIG2;GALNT1;SEL1L;ELOVL6;PLOD2;HSP90B1*	29
	G2-M Checkpoint	*TOP2A;CDKN2C;DBF4;KIF11;HMMR;MCM6;LBR;BUB1;SAP30;LMNB1*	15
	Golgi membrane (GO:0000139)	*GALNT7;GALNT1;GCNT1;RHOBTB3;MANEA;SORL1;HS2ST1;UGCG;GJA1;C1GALT1C1*	23
	Intrinsic apoptotic signaling pathway (GO:0097193)	*ERO1A;BNIP3;CD24;PDK1;MLLT11;PRKDC;BNIP3;ARL6IP5;ATM;CD24*	11
	Mitotic cell cycle phase transition (GO:0044772)	*CCNB1;CDKN2C;BORA;DBF4;CCNE2;PRKAR2B;CCNG2;CEP70;SKP2;AURKA*	19
	Mitotic Spindle	*TOP2A;ANLN;KIF11;DOCK2;BUB1;DLGAP5;LMNB1;AURKA;DYNC1H1;CENPE*	13
	mTORC1 Signaling	*ERO1A;ELOVL6;PLOD2;VLDLR;ADD3;SYTL2;HSP90B1;AURKA;LDHA;P4HA1*	26
	Protein digestion and absorption	*COL3A1;COL5A1;MME;COL11A1;COL8A1;SLC8A1;COL3A1;COL11A1;COL5A2;COL4A5*	10
	UV Response Dn	*PTGFR;GJA1;COL3A1;PRKAR2B;COL11A1;ID1;GCNT1;VLDLR;TFPI;ADD3*	22

Figure S1. The original western blot images.

GADD45B

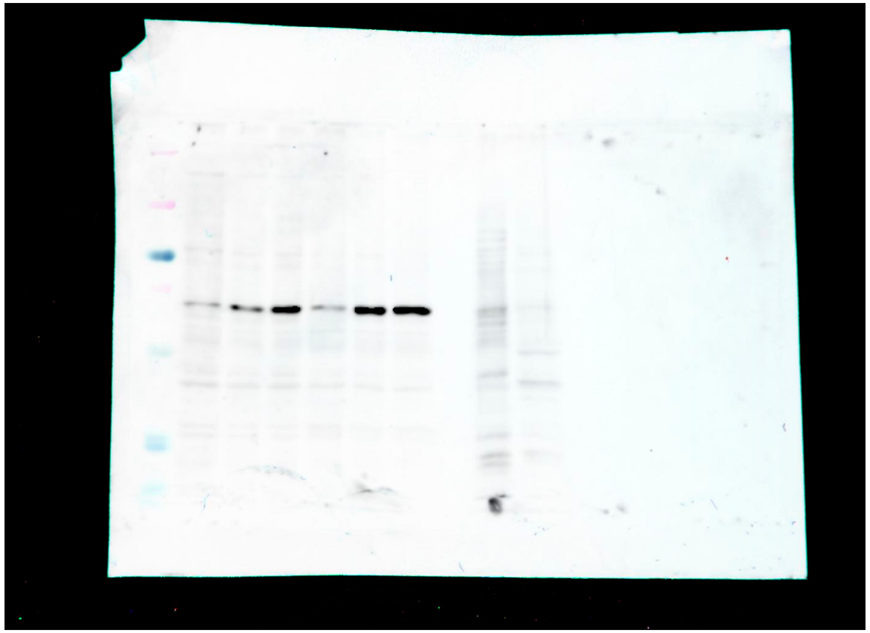

ATF3

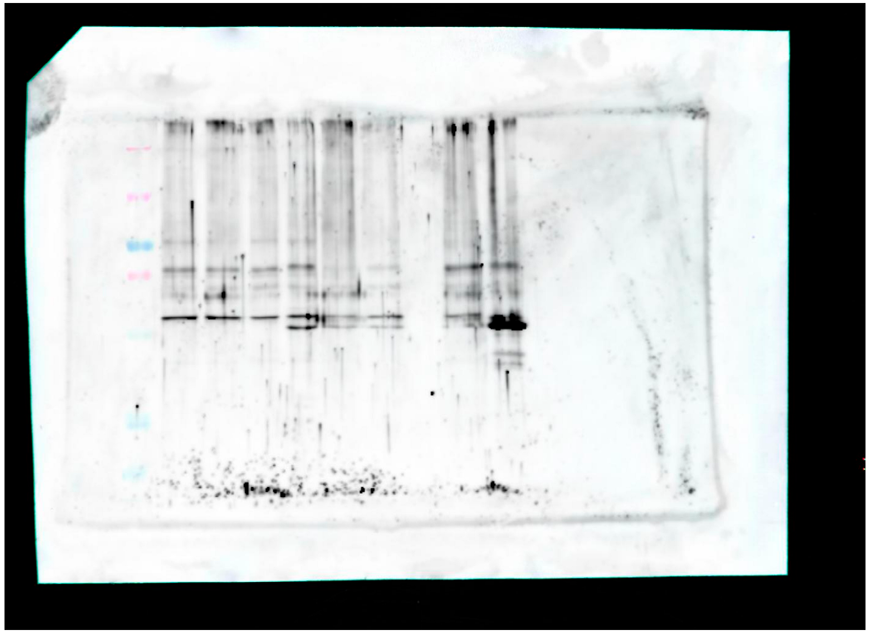

Actin + GADD45

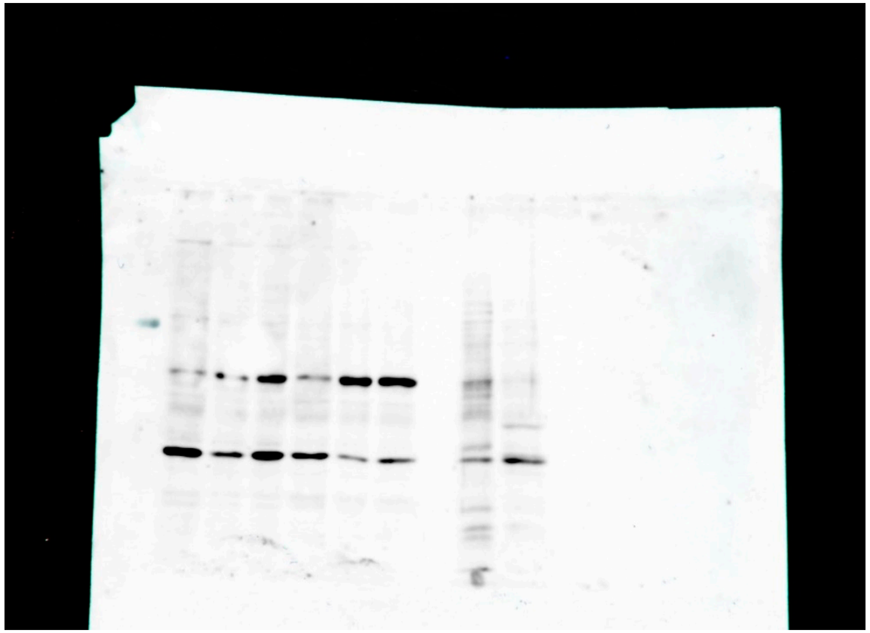

γH2AX

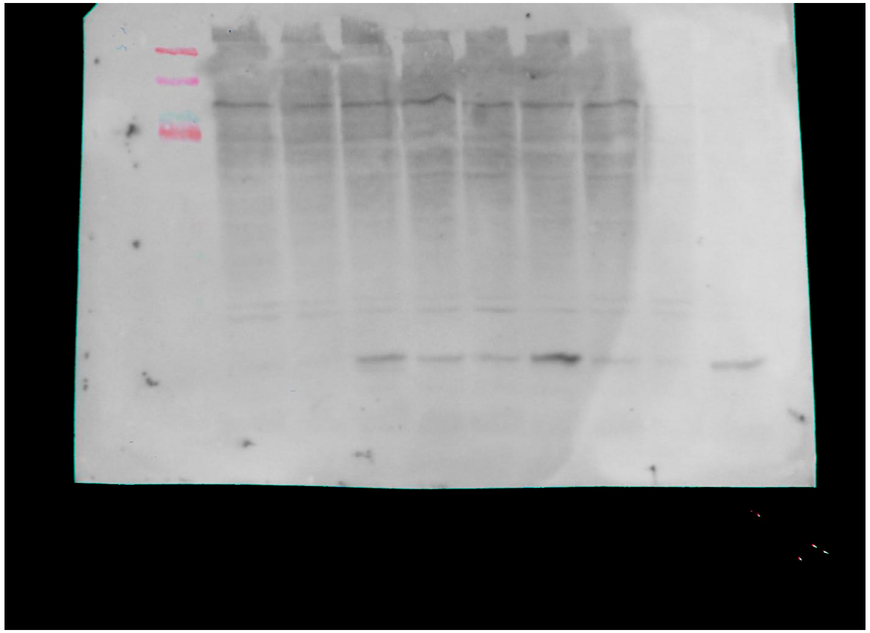

MDM2

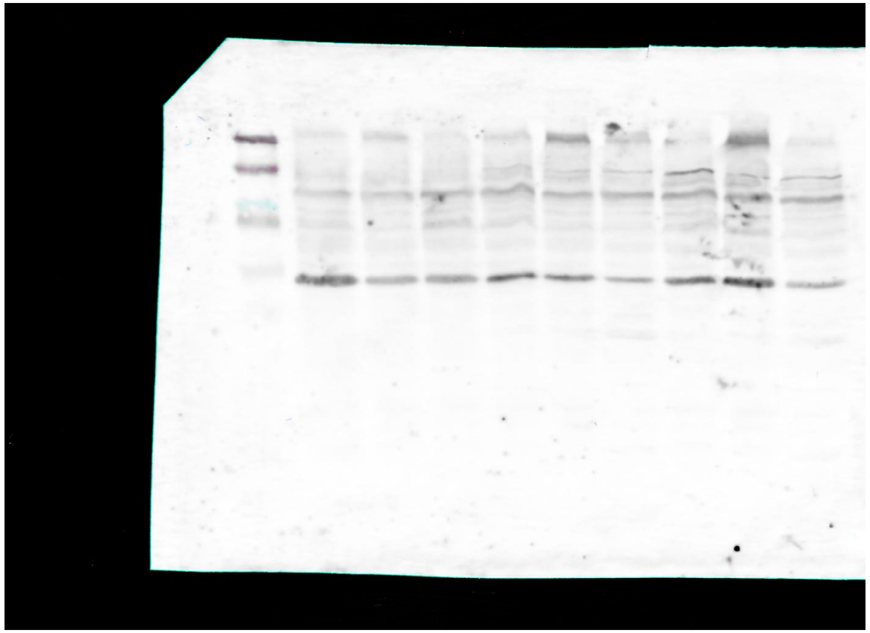

β-Tubulin

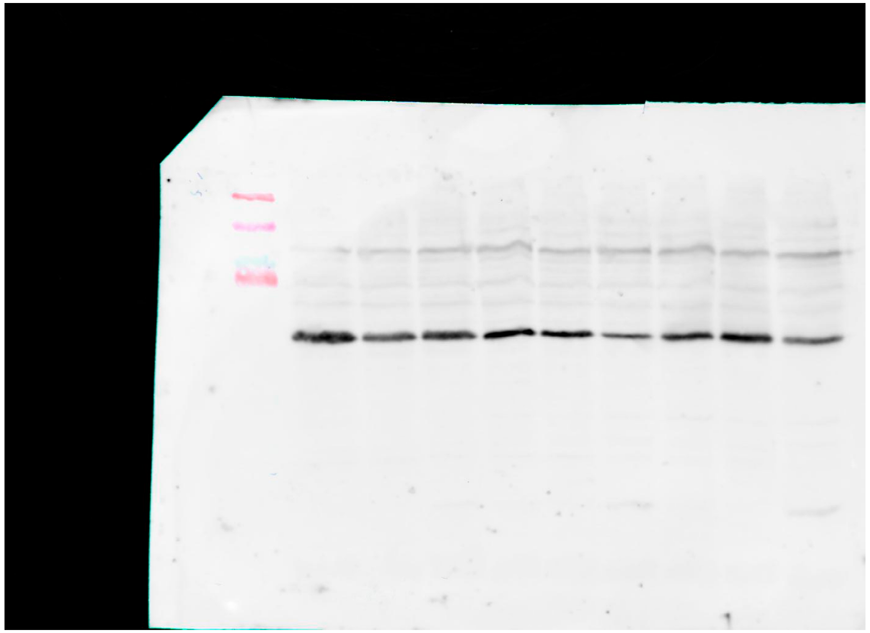

GPX4

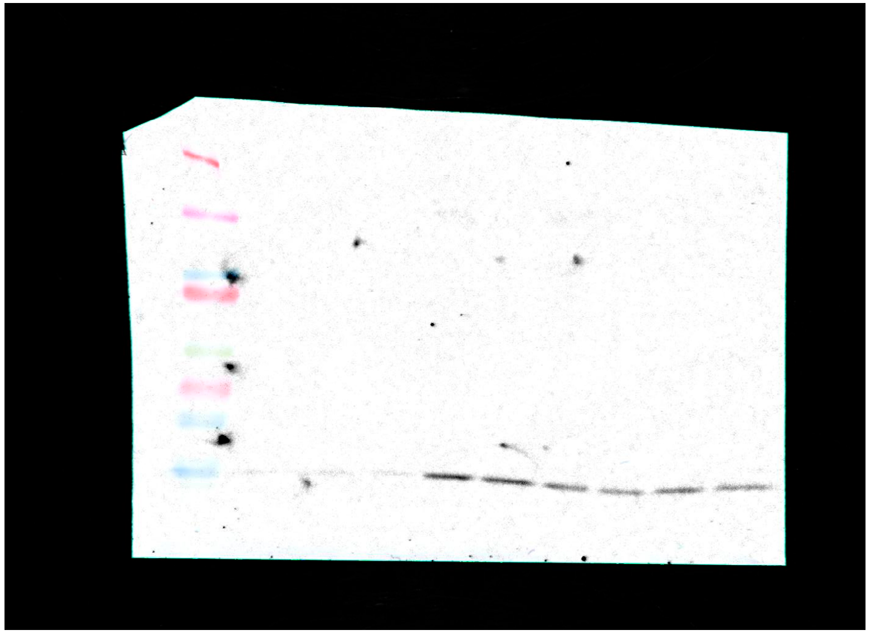

GPX7 + Tubulin

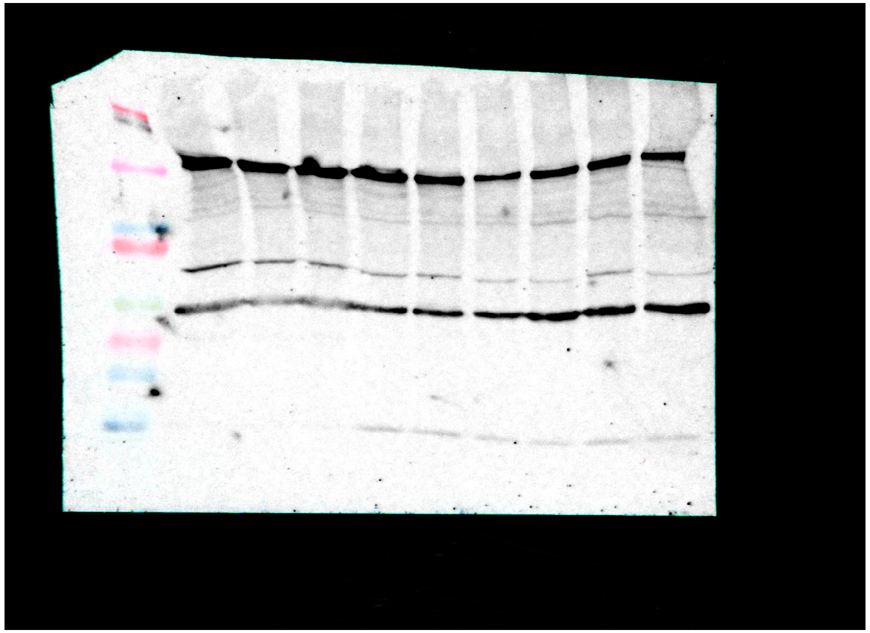

Tubulin

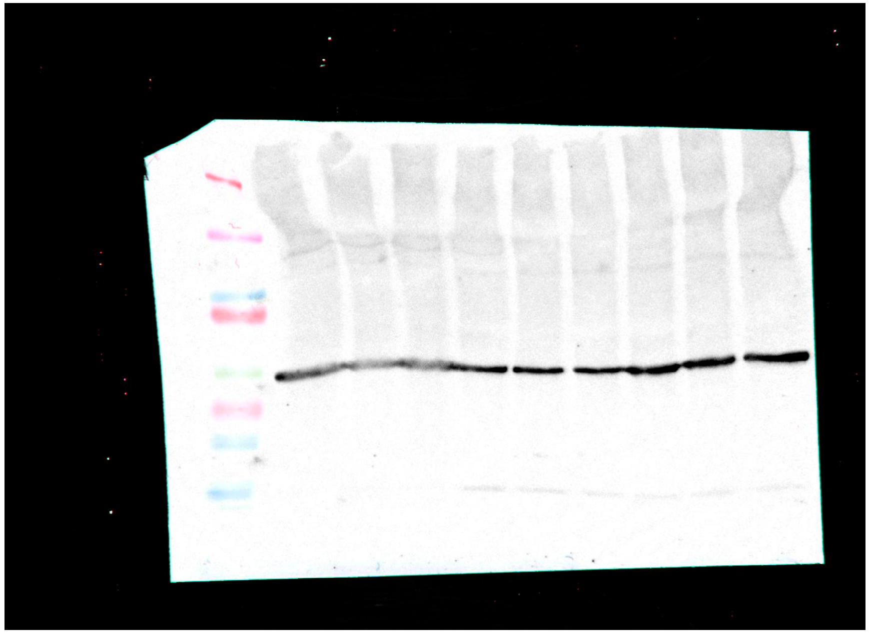

Supplement: Supplementary file 1 [file biomolecules-13-01672-s001.zip › Supplementary matherials.pdf]
